# Former APS Clients’ Perspectives on Barriers to Safe and Stable Housing

**DOI:** 10.1093/geroni/igaf122.4122

**Published:** 2025-12-31

**Authors:** Mia Canzone, Samantha Tuft, Courtney Reynolds

**Affiliations:** Benjamin Rose, Cleveland, Ohio, United States; Benjamin Rose, Cleveland, Ohio, United States; Benjamin Rose, Cleveland, Ohio, United States

## Abstract

Oklahoma Adult Protective Services (APS) partnered with Benjamin Rose to conduct a needs assessment to better understand/serve APS clients needing safe housing. Ten former APS clients participated in semi structured interviews to educate Benjamin Rose and APS about their housing searches. Fifty percent of participants identified as male (n = 5). Average age recorded was 59 years old (Range = 30-76). Racial backgrounds included White (n = 6), American Indian/Alaska Native (n = 4), and Black/African American (n = 3); 40% identified as multiracial. Participants resided in apartment complexes/homes (n = 7), an assisted living facility (n = 1), or were unhoused (n = 2). Most described homelessness as a series of unfortunate incidents. Preliminary results included themes of inaccessible housing (“I have a disability of course, and that requires me to use a walker. . . I can’t just go pick a house or an apartment that I want that’s within my budget . . .”), insufficient emergency housing infrastructure (after living in a mental hospital, hospital, rehab, and assisted living facility, one participant stated, “. . . it was better than being on the street in a tent”), and perceived lack of APS involvement (“I feel like the whole APS is a liar because, they will not find me a place to live”). These findings are informing the development of an innovative elder shelter program for APS clients in need of safe housing. A major implication includes the need for a systemic approach to supporting unhoused APS clients.

